# Prevalence of COVID-19 in children affected by allergic rhinoconjunctivitis and asthma: results from the second “SIAIP rhinosinusitis and conjunctivitis committee” survey

**DOI:** 10.1186/s13052-021-01198-y

**Published:** 2022-01-06

**Authors:** Giulia Brindisi, Anna Maria Zicari, Giuseppe Fabio Parisi, Lucia Diaferio, Cristiana Indolfi, Giuseppe Marchese, Daniele Giovanni Ghiglioni, Giuseppina Rosaria Umano, Angela Klain, Gian Luigi Marseglia, Michele Miraglia Del Giudice

**Affiliations:** 1 Department of Maternal Infantile and Urological Sciences, Division of Pediatric Allergology and Immunology, Sapienza University of Rome, 00185 Rome, Italy; 2 Department of Clinical and Experimental Medicine, University of Catania, Via Santa Sofia 78, 95123 Catania, Italy; 3 Department of Pediatrics, Giovanni XXIII Hospital, University of Bari, 70126 Bari, Italy; 4 Department of Woman, Child and Specialized Surgery, University of Campania “Luigi Vanvitelli”, Via L. De Crecchio 2, 80138 Naples, Italy; 5 Primary care pediatrician, Cedegolo, Brescia, Italy; 6 Fondazione IRCCS Ca’ Granda Ospedale Maggiore Policlinico di Milano, Via Francesco Sforza, 28, 20122 Milan, Italy; 7 Department of Pediatrics, Foundation IRCCS Policlinico San Matteo, University of Pavia, Viale Camillo Golgi 19, 27100 Pavia, Italy

**Keywords:** SARS CoV2, COVID - 19, Children, Asthma, Allergic rhinoconjunctivitis, Online-survey

## Abstract

**Background:**

The role of allergic sensitization seems to be protective against SARS CoV2 infection. The aim of this study was to evaluate, using online surveys, the impact of COVID-19 on Italian allergic children, comparing the prevalence of AR and asthma symptoms between the first and second pandemic wave.

**Methods:**

Both surveys were emailed to Italian pediatricians in April 2020 (first survey) and in March 2021 (second survey). The first one was related to the impact of COVID-19 and the most frequently reported symptoms. The second one was superimposed on the previous one, taking into account some additional aspects in the management of disease.

**Results:**

A total of 99 pediatricians participated in the first survey and 267 in the second one. The first survey showed that, asthma and allergic rhinoconjunctivitis prevalence was mostly between 0 and 20% throughout the country. The second survey showed a lower prevalence of both diseases nationwide in comparison to the first one. Comparing the two surveys, statistically significant differences were reported only in the distribution of asthma prevalence in Southern Italy while no differences were highlighted in the North and in the Center. Finally regarding allergic rhinoconjunctivitis prevalence, no differences were noticed nationwide.

**Conclusions:**

Allergic rhinoconjunctivitis and asthma, if under control, did not represent risk factors for the susceptibility to SARS CoV2. Therefore, it is strongly recommended to continue therapies during COVID-19 outbreak, according to the international guidelines. However, being COVID-19 a new disease, actual knowledge will undergo continuous improvements over time.

## Background

The current outbreak of novel coronavirus disease 2019 (COVID-19), declared pandemic in March 2020 by the World Health Organization (WHO) has caused millions of deaths all over the world [1]. Italy is considered one of the most hit countries, with 4.248.225 reported cases and 126.758 deaths, at the time of this writing [2].

Although in adulthood the rate of symptoms severity and mortality is higher than in children, the appearance of several “severe acute respiratory syndrome coronavirus 2 (SARS CoV2)” variants has made the pediatric population more susceptible to COVID-19 compare to the beginning of the pandemic [3].

Common symptoms in children are fever, gastrointestinal and skin manifestations, arthralgia, anosmia and/or ageusia, rhinorrhea, cough and dyspnea that only rarely require hospitalization in intensive care units [4, 5].

Allergic rhinitis (AR) and asthma are common allergic diseases, often associated with each other for the anatomical continuity between upper and lower airways. Furthermore, they share the same inflammatory pathway, characterized by a T helper 2(Th2) response with the presence of several biomarkers accountable for the characteristic chronic inflammation throughout the bronchial tree [6, 7].

As both AR and asthma can present an overlap with COVID-19, a careful monitoring of these symptoms and a prompt differential diagnosis is desirable [8].

To date, the role of allergic sensitization has been reconsidered in relation to COVID-19, no longer as a risk factor but on the contrary as a protective one against SARS CoV2 infection in several studies. Du H et al. analyzed 182 children hospitalized for COVID-19 of whom 23.6% had allergic disease and only one child of these reported asthma. The findings of this study showed no statistically significant differences in disease severity, development of complications, and clinical symptoms between the allergic and non-allergic group [9].

Many are the possible explanations for not considering allergy as a risk factor for SARS CoV2 infection. One of them is the protective role of eosinophils and the related Th2 phenotype associated with allergy, with a low Interferon-α (IFN-α) production; in fact it seems that the higher the eosinophil count is, the better the prognosis of COVID-19 is with reduced morbidity and mortality [10].

Additionally, respiratory allergies are associated with a reduced expression of angiotensin conversion enzyme receptor 2 (ACE2), the well-known entrance door of SARS CoV2 into the upper airways cells. Th2 response is able to modulate ACE2 expression with an intriguing role in COVID-19 pathogenesis, acting on the Th1/Th2 balance [11]. Therefore it seems that Th2 inflammation exerts a protective role against SARS CoV2 [12].

Lastly, several studies show that asthma medications such as inhaled steroids, bronchodilators and montelukast, present an antiviral and immunomodulatory effect that inhibits SARS CoV2 driven inflammation [13].

In line with the scientific literature, data derived from the first survey on the impact of COVID-19, realized by the “SIAIP rhinosinusitis and conjunctivitis committee”, concluded that allergic rhinoconjunctivitis and asthma were not risk factors for the development of severe forms of COVID-19 [14]. On the other hand, the presence of uncontrolled allergic symptoms can represent a marker of poor prognosis and disease severity [15].

Therefore, the achievement of both AR and asthma control appears fundamental not only in the daily clinical practice [16] but above all during pandemic [15].

Following the first survey, related to the period of December–May 2020, the “SIAIP rhinosinusitis and conjunctivitis committee” decided to issue a new questionnaire referring to the period of September–February 2021, aiming to assess nationwide the impact of COVID-19 among children and the presence of any differences compared to the first pandemic peak.

By analyzing the above mentioned surveys in this study we aimed to compare the prevalence of allergic rhinoconjunctivitis and asthma symptoms among children affected by COVID-19 in the North, Center and South of Italy.

## Methods

### Study design and population

The first “COVID-19 survey” was sent by email to Italian pediatricians in April 2020 and was composed of 20 multiple choice questions, related to the impact of COVID-19, the disease’s management and the most frequently reported symptoms [17].

Then in March 2021 the second “Follow-up COVID-19 survey” was emailed to a greater number of Italian pediatricians and was composed by 35 multiple choice questions; it was superimposed on the previous one, by taking into account some additional aspects in the management of disease.

Both questionnaires were created by the “SIAIP rhino-sinusitis and conjunctivitis committee” in Italian, using Google-Drive Platform (Google DriveTM,© 2012 Google Inc. all rights reserved).

The anonymous data received were automatically collected in an Excel© data format used for the statistical analysis. Both surveys took approximately 10 min to be answered. Each pediatrician was allowed to answer only once to avoid double responses. There was no need to obtain informed consent, given the voluntary nature of both surveys. The Ethics Committee of Sapienza University of Rome, did not consider any special permission necessary because these studies’ designs met the criteria of an audit activity.

### Statistical analysis

All the variables were categorical. Differences were investigated by Chi square test. Data were presented as frequency. All analyses were performed using SAS University Edition (SAS Institute, Cary, NC, USA), and statistical significance was assessed at the two-tailed 0.05 threshold.

## Results

The already published data related to the first survey collected a total of 99 responses of which 32% from the North of Italy, 27.8% from the Center and 40.2% from the South [16].

A total of 267 responses were derived from the second survey, of which 53.2% from the North, 27.4% from the Center and 19.4% from the South. Characteristics of the pediatricians who responded to both surveys are shown in Tables 1 and 2.

**Table 1:** First Survey Participants’ characteristics [16]

Total of participants	99
**Types of pediatricians**	
Primary care	60%
Pediatric hospital medicine	24.2%
Specialized outpatient healthcare	6.3%
Pediatric emergency medicine	6.3%
Pediatric critical care medicine	2.1%
Pediatric infectious disease	1.1%
**Territorial Subdivisions**	
North	32%
Center	27.8%
South an Islands	40.2%

**Table 2:** Second Survey Participants’ characteristics

Total of participants	267
**Types of pediatricians**	
Primary care	75.5%
Pediatric hospital medicine	16.3%
Specialized outpatient healthcare	5.4%
Pediatric emergency medicine	1.9%
Pediatric critical care medicine	0.9%
Pediatric infectious disease	0%
**Territorial Subdivisions**	
North	53.2%
Center	27.4%
South and Islands	19.4%

Analyzing the first survey, we found statistically significant differences for asthma prevalence among children affected by COVID-19 considering the different zones of Italy (*p* = 0.03).

**Fig. 1:**
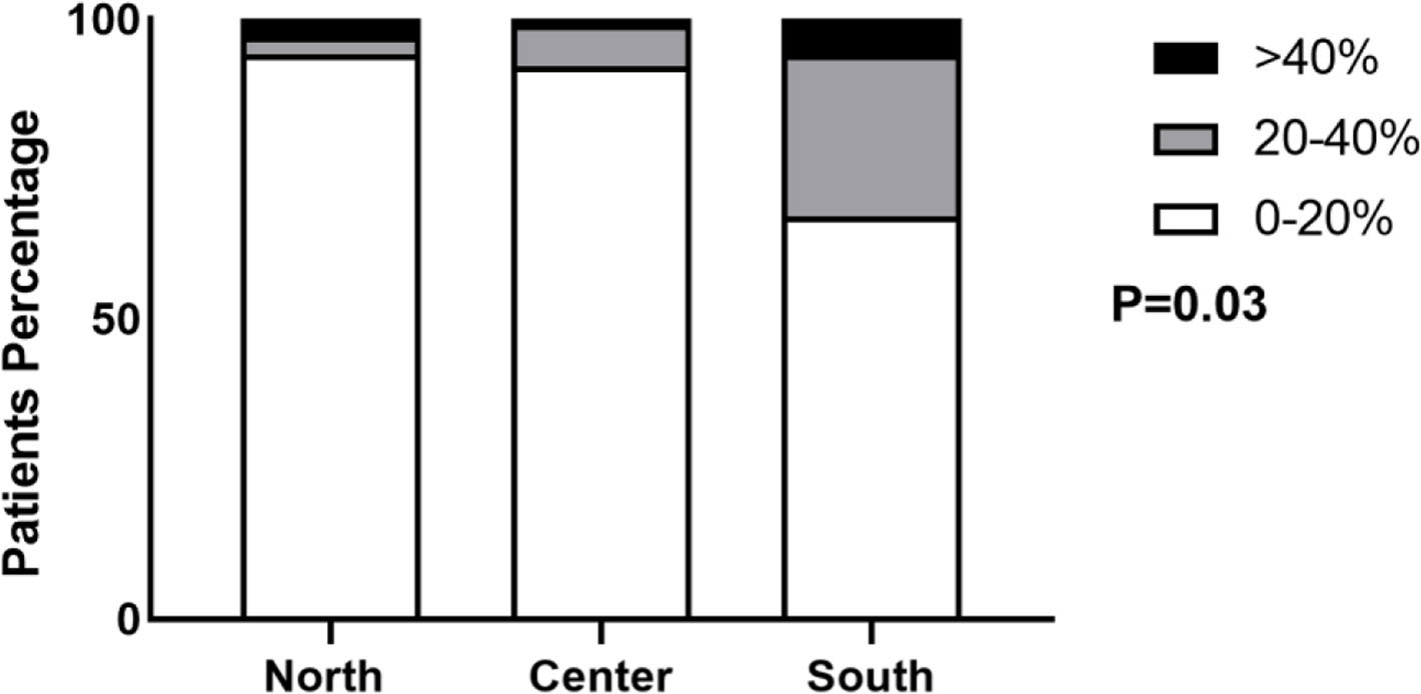
Prevalence of asthma by geographic area during the first survey

In detail in the North, 94% of children presented a prevalence less than 20, 3% between 20 and 40 and 3% greater than 40%. In the Center, 92% of children featured a prevalence less than 20, 8% between 20 and 40% and no one greater than 40%. Lastly in the South, 67% of children showed a prevalence less than 20, 27% between 20 and 40 and 6% greater than 40%. In particular, the South of Italy showed the highest rate of asthma prevalence compared to the North and Center (Fig. 1).

Prevalence of asthmatic patients according to geographic area during the first COVID-19 wave. Data are expressed as percentage. White bar refers to < 20% prevalence, green to 20–40% prevalence, and black to > 40% prevalence.

Also, the second survey showed statistically significant differences (*p* = 0.03) for asthma prevalence in the considered zones of Italy, as following: the Center had the same prevalence of the South, whereas the distribution in the North did not change significantly in comparison to the first survey.

**Fig. 2:**
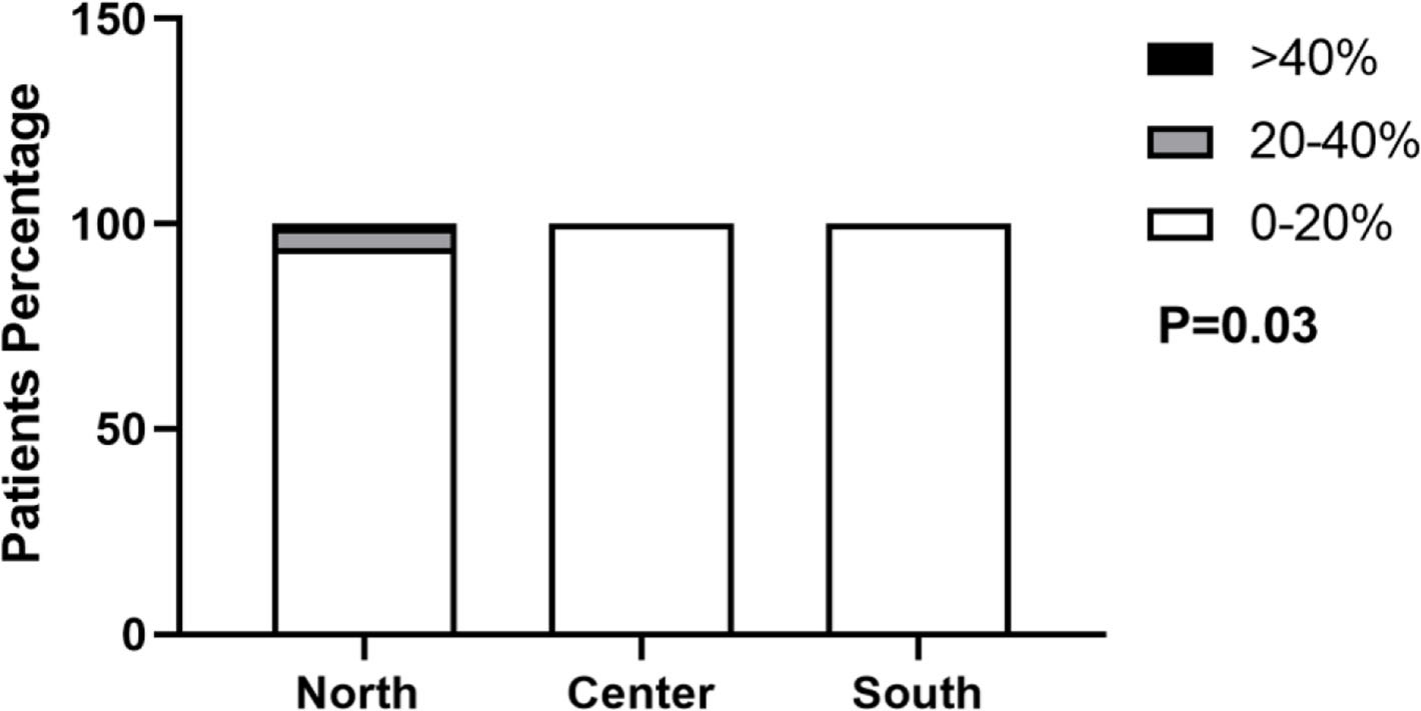
Prevalence of asthma by geographic area during the second survey

In particular, the North presented a prevalence less than 20% in 94% of children, between 20 and 40% in 5% and greater than 40% in 1%. In the Center and South, the totality of affected children (100%) presented a prevalence less than 20% (Fig. 2).

Prevalence of asthmatic patients according to geographic area during the second COVID-19 wave. Data are expressed as percentage. White bar refers to < 20% prevalence, green to 20–40% prevalence, and black to > 40% prevalence.

**Fig. 3:**
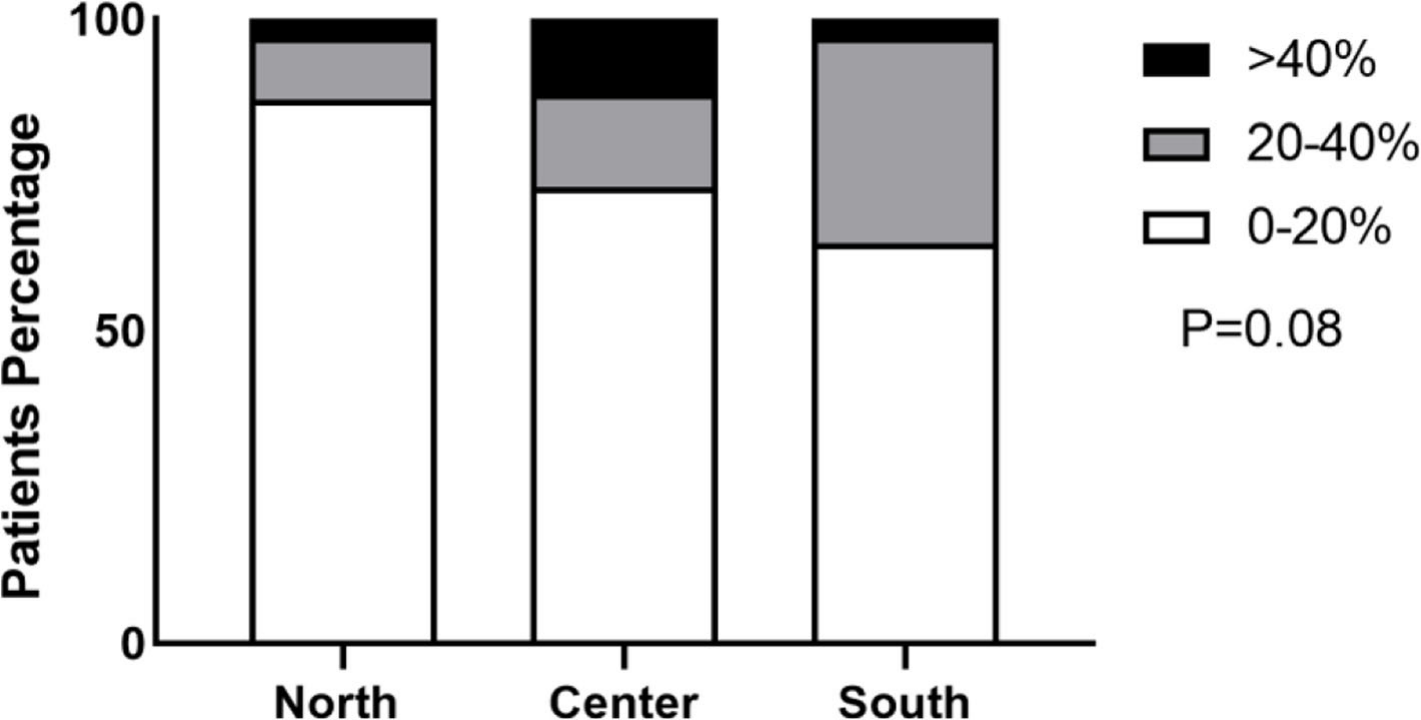
Prevalence of allergic rhinoconjuntivitis by geographic area during the first survey

Considering symptoms of allergic **r**hinoconjuntivitis**,** in the first survey we observed a trend for a higher rate of symptoms in the Center and South of Italy, however these differences did not reach statistically significance (*p* = 0.08). The North showed a prevalence less than 20% in 87% of children, between 20 and 40% in 10% and greater than 40% in 3%. In the Center, 73% of children reported a prevalence less than 20, 15% between 20 and 40 and 12% greater than 40%. Lastly in the South, 64% reported a prevalence less than 20, 33% between 20 and 40% and only 3% greater than 40% (Fig. 3).

Prevalence of allergic rhinoconjutivitis according to geographic area during the first COVID-19 wave. Data are expressed as percentage. White bar refers to < 20% prevalence, green to 20–40% prevalence, and black to > 40% prevalence.

**Fig. 4:**
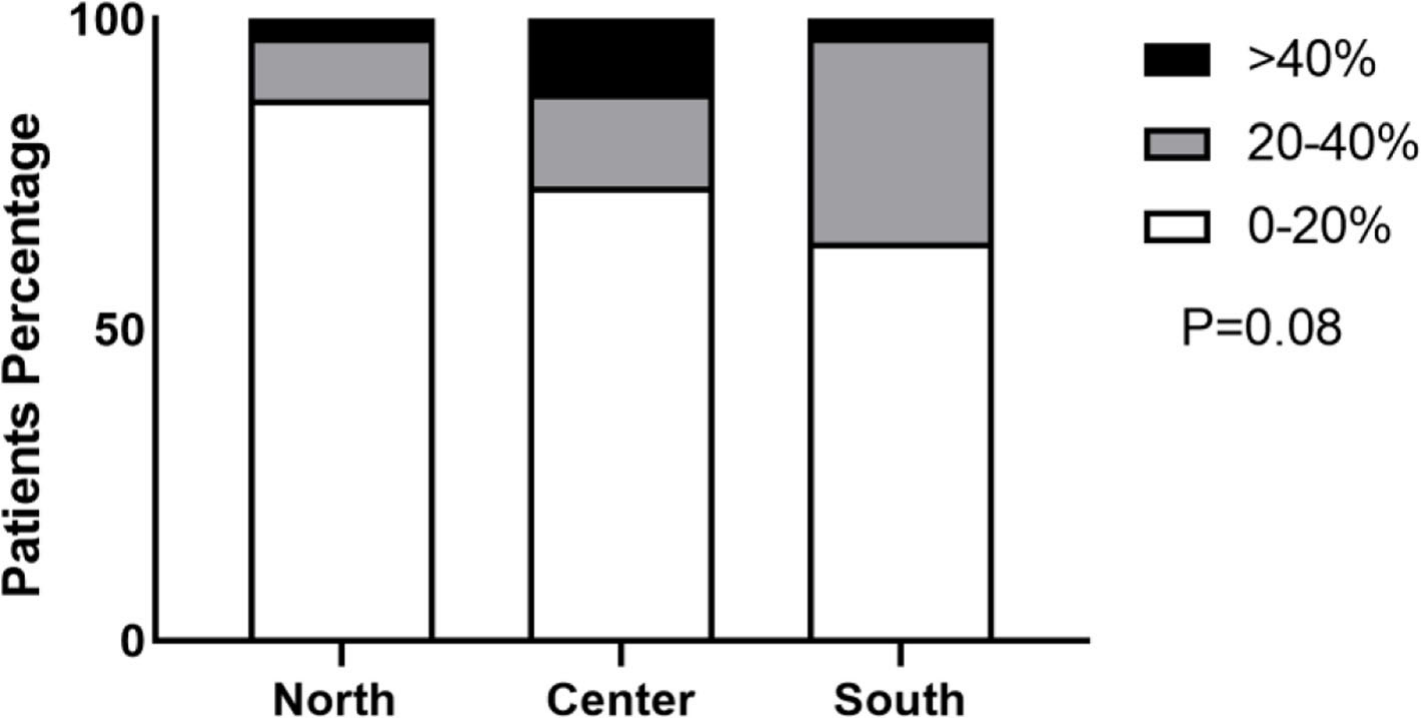
Prevalence of allergic rhinoconjuntivitis by geographic area during the second survey

In the second survey, Center and South Italy showed significant higher rates of allergic rhinoconjuntivitis compared to North (*p* = 0.049) where 89% of children reported a prevalence less than 20, 10% between 20 and 40% and no one greater than 40%. In the Center, 84% showed a prevalence less than 20, 14% between 20 and 40 and 2% greater than 40%. Lastly in the South, 75% of children had a prevalence less than 20, 24% between 20 and 40% and only 1% greater than 40% (Fig. 4).

Prevalence of allergic rhinoconjutivitis according to geographic area during the second COVID-19 wave. Data are expressed as percentage. White bar refers to < 20% prevalence, green to 20–40% prevalence, and black to > 40% prevalence.

Furthermore comparing the two surveys, statistically significant differences were reported only in the distribution of asthma prevalence in the South (*p* < 0.0001) while no differences were highlighted in the North and in the Center (*p* > 0.05); the first survey showed a higher rate of asthma compared to the second one. Finally regarding allergic rhinoconjuntivitis distribution, no differences were noticed nationwide (*p* > 0.05).

## Discussion

Allergy does not appear to be a predisposing factor for the development of SARS CoV-2 infection or an aggravating one in case of disease [17].

Dong et al., analyzing children and adults affected by COVID-19, reported that no patients with allergies presented critical symptoms or had a more severe course of illness than those without allergies. All this is in line with the hypothesis of a positive role exerted by the Th2 immune response in COVID-19 pathogenesis [18].

Kimura et al., highlighted that IL-4 and IL-13, typical cytokines of a Th2 response, down-regulating ACE2 receptors in upper airways cells of allergic children, would exert protection against SARS CoV2 infection; otherwise the Th1 response, with interferon-gamma (IFN-gamma) release, increases ACE2 expression, promoting SARS CoV-2 entry into airway cells. All these observations, confirm the role played by the Th1/Th2 balance in the pathogenesis of COVID-19 [19].

The systematic review of the literature, conducted by Morais-Almeida et al., supports these results, analyzing the relationship between asthma and COVID-19 in several studies and highlighting no evidence of a higher risk of being infected by SARS CoV2 or to develop a critical illness among asthmatic patients [20].

Hence, according to our results, children with asthma and allergic rhinoconjutivitis present a low prevalence of SARS CoV2 infection, because the ACE2 receptors used by the virus to enter the respiratory tract are less expressed in allergic phenotypes. Conversely, other conventional coronaviruses or respiratory viruses are able to exacerbate asthma because they use other entry receptors different to ACE2 [21].

Moreover, these ACE2 are also down-regulated by asthma therapies that must be continued during the outbreak, according to the international guidelines, to obtain the control of allergic symptoms, also achieved respecting the current social distances and public hygiene recommendations [15].

A good COVID-19 prognosis is also linked to a high blood eosinophil count, common finding in allergic diseases in the context of a Th2 response [20]. Du Y et al., reported that eosinopenia was found in about 80% of patients died for COVID-19, considering it as a biomarker of worse prognosis and a predictor for the development of COVID-19 severity [22].

So, although asthma and allergic symptoms are often associated to COVID-19 they did not represent risk factors for a more severe disease course and did not lead to a higher frequency of hospitalization [23]. These results were also confirmed in a wide online survey conducted on 133.000 asthmatic children who reported mild symptoms and only one case of hospitalization for COVID-19 [24].

Moreover, during the pandemic, the lower prevalence of asthmatic and allergic exacerbations is also attributable to greater public health hygiene measures, the use of facemasks, the lockdown periods and a prompter management of respiratory symptoms by parents [25].

Obviously, symptoms of uncontrolled asthma and allergic symptoms, are considered risk factors for the development of severe COVID-19 [15] .

For this reason, the Global Initiative for Asthma (GINA) claimed the necessity to achieve asthma control by continuing, even during the pandemic, therapies including biologic drugs, except in patients with active COVID-19 [26–28].

However, in addition to the management of asthma, also AR requires to be adequately treated [7] because it may have an overlap of symptoms with COVID-19 infectious rhinitis. Uncontrolled AR can increase the risk of viral transmission in children with COVID-19. ARIA and EAACI reiterated the importance of performing a correct nasal therapy with intranasal corticosteroid to avoid the worsening of allergic symptoms and, in case of SARS CoV2 infection, the viral spread [29].

On the other hand, it is recommended to discontinue allergen immunotherapy (AIT), as it decreases the TH2 response, in COVID-19 patients and to continue it in allergic patients without the infection [30, 31].

Summarizing, allergic patients present a Th2 response with a lower production of IFN-α in addition to the presence of eosinophils and low ACE2 expression in the airway tact; in addition, asthmatic patients benefit from the antiviral/ immunomodulatory effect exerts by asthma medications [13].

All these factors act as a protective mechanism, reducing the hyperinflammation triggered by the virus to which the more severe clinic is linked.

In line with the literature, the results of both surveys, provide data on the prevalence of allergic rhinoconjuntivitis and asthma symptoms in children affected by COVID-19 nationwide, supporting the concept that controlled allergic symptoms do not constitute risk factors for SARS CoV2 susceptibility.

These results, obtained by the preliminary analysis of the first COVID-19 survey [14], were also confirmed by the second one. In particular, the first survey showed that, in relation to both asthma and allergic rhinoconjunctivitis, the general prevalence was mostly between 0 and 20% throughout the country. All in all, northern regions showed a lower prevalence of allergic symptoms than the Center and South.

The second survey highlighted a lower prevalence of asthma nationwide in comparison to the first one; in particular it confirmed that the North had the lowest prevalence of both asthma and allergic rhinoconjunctivitis and it reported a marked improvement in the prevalence of both diseases in the Center and South.

Comparing the two surveys, statistically significant differences were reported only in the distribution of asthma prevalence in Southern Italy, while no differences were found in the North and in the Center. Lastly regarding allergic rhinoconjunctivitis distribution, no differences were noticed at a national level.

In support of our findings, another Italian survey conducted among children with AR and/or asthma to evaluate allergic symptoms and the need of drug usage during lockdown, showed an overall trend of clinical improvement and a reduction of on demand and basal therapy for both AR and asthma. All these results can be attributable to various factors: the prompt management of allergic symptoms by parents and the minor exposure to pollens, viruses, and air pollution due to the lockdown [25].

Similar results achieved by the already mentioned survey conducted by Papadopoulos et al., confirmed that children with asthma were not affected to a great extent by COVID-19, due to better adherence to preventive hygiene measures and improved treatment compliance [24].

This study presents also some limitations. First of all, these two surveys refer to slightly different periods, enrolling a different number of participants. Furthermore, we used non standardized questionnaires that were filled out by pediatricians from different parts of Italy, carrying out heterogeneous activities among all of them. Anyway, to the best of our knowledge, standardized and validated questionnaires on these topics are not currently available.

## Conclusions

Asthma and allergic rhinoconjunctivitis, if under control, did not represent risk factors for SARS CoV2 susceptibility or severity. Therefore, the need to continue both on demand and basal therapy during COVID-19 outbreak, according to the international guidelines. However, being COVID-19 a new disease, we expect that actual knowledge will undergo continuous improvements over time.

## Data Availability

The datasets used and/or analysed during the current study are available from the corresponding author on reasonable request.
